# Mitochondrial transplantation reverses the senescence phenotype of SH-SY5Y cells

**DOI:** 10.1016/j.omta.2026.201788

**Published:** 2026-06-17

**Authors:** Liqun Xu, Yilang Wu, Wanfei Wu, Xiao Li, Ronghao Deng, Haibao Zhu, Aihua Mao, Pingnan Sun, Xin Zhang, Wencan Xu, Chi-ju Wei

**Affiliations:** 1 Guangdong Provincial Key Laboratory of Marine Biotechnology, Institute of Marine Sciences, Shantou University, Shantou 515063, Guangdong, China; 2 Stem Cell Research Center, Shantou University Medical College, Shantou, Guangdong 515041, China; 3 Laboratory of Molecular Cardiology, The First Affiliated Hospital of Shantou University Medical College, Shantou, Guangdong 515041, China; 4 Department of Endocrinology, The First Affiliated Hospital of Shantou University Medical College, Shantou, Guangdong 515041, China

**Keywords:** plasma membrane vesicles, PMVs, Parkinson disease, mitochondria transplantation, autophagy, single-nucleotide polymorphism analysis, SNP analysis, cellular senescence, SH-SY5Y cells

## Abstract

Fusogenic plasma membrane vesicles (PMVs) were engineered as carriers for mitochondrial delivery into senescent SH-SY5Y cells, a human neuroblastoma cell line widely used as an *in vitro* model for neurodegenerative diseases. Mitochondrial transfer was achieved via cell fusion mediated by the fusogenic vesicular stomatitis virus glycoprotein G. After mitochondrial transplantation, senescent SH-SY5Y cells exhibited marked phenotypic reversal, accompanied by restoration of glucose metabolism, ATP production, lactate levels, and mitochondrial respiratory activity to near-normal levels. In addition, mitochondrial transplantation regulated the senescence-associated secretory phenotype and associated inflammatory signaling pathways, while significantly enhancing antiapoptotic activity. Single-nucleotide polymorphism tracing of mitochondrial DNA confirmed the stable persistence of transplanted mitochondria within recipient cells, which was associated with recovery of normal mitochondrial morphology, function, and biogenesis. Notably, autophagic activity decreased after mitochondrial transplantation. Finally, alpha-synuclein expression was reduced, whereas dopamine production and the activities of enzymes involved in dopamine synthesis were increased after mitochondrial transplantation. The results demonstrated that mitochondrial transplantation can effectively reverse the senescence phenotype of SH-SY5Y cells, suggesting that mitochondrial transplantation may represent a promising therapeutic strategy for neurodegenerative disorders such as Parkinson disease.

## Graphical abstract

**Figure:**
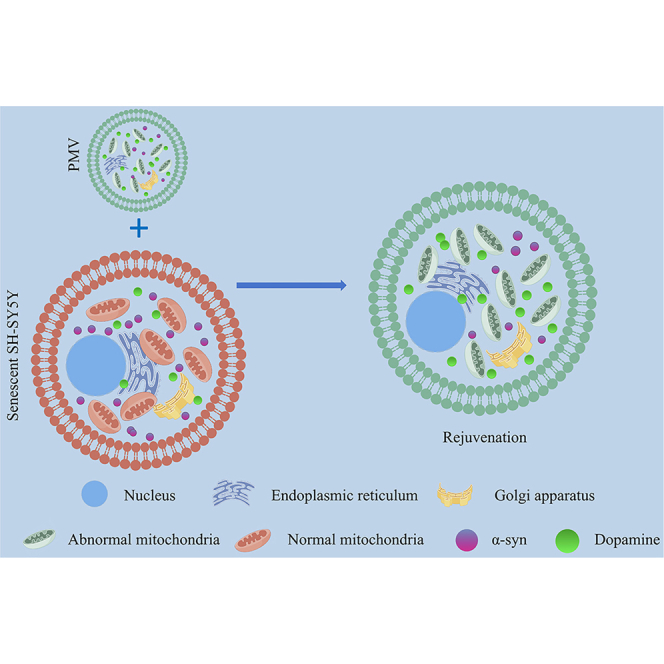

## Teaser

Mitochondrial dysfunction represents a pivotal characteristic of numerous neurodegenerative disorders. This article describes an approach of mitochondrial transplantation using plasma membrane vesicles, which are able to reverse the senescence phenotype of SH-SY5Y cells, decrease the expression of α-synuclein, and increase the production of dopamine and related enzymatic activities.

## Introduction

Mitochondrial dysfunction is a hallmark of numerous neurodegenerative disorders, including both genetic and sporadic forms of Parkinson disease (PD).^1^ Impaired energy metabolism, excessive generation of reactive oxygen species (ROS), and defective mitochondrial quality control are frequently observed before the onset of overt neurodegeneration.^2^^,^^3^^,^^4^ Studies using animal models of PD have demonstrated that the neurotoxin MPTP (1-methyl-4-phenyl-1,2,3,6-tetrahydropyridine) induces mitochondrial damage in dopaminergic neurons by inhibiting complex I (CI) of the electron transport chain.^5^^,^^6^ Dysfunctional mitochondria further exacerbate oxidative stress, impair calcium buffering capacity, and dysregulate proapoptotic signaling pathways, thereby creating a self-perpetuating cycle of mitochondrial dysfunction and progressive neuronal degeneration.

Neuropathologically, PD is characterized by selective degeneration of dopaminergic neurons in the substantia nigra pars compacta (SNc) and by the accumulation of alpha-synuclein (αSyn) aggregates, known as Lewy bodies.^7^ Extensive experimental evidence indicates that αSyn pathology is a major contributor to mitochondrial dysfunction.^8^^,^^9^^,^^10^ Under physiological conditions, αSyn can translocate to mitochondria and modulate mitochondrial ATP synthase activity.^11^ However, αSyn overexpression reduces mitochondrial CI activity and mitochondrial membrane potential, while simultaneously increasing oxidative stress.^12^^,^^13^^,^^14^

Mitochondria-targeted therapy is an attractive strategy for the treatment of PD.^15^^,^^16^ Clinical studies investigating compounds that target mitochondrial oxidative stress, metabolism, mitochondrial biogenesis, and calcium homeostasis in patients with idiopathic PD have demonstrated promising neuroprotective and symptomatic benefits.^17^ Antidiabetic agents such as exenatide and pioglitazone reduce PD risk by suppressing neuroinflammatory pathways, decreasing ROS production, lowering intracellular Ca^2+^ levels, restoring mitophagy, improving bioenergetic efficiency, and promoting mitochondrial biogenesis.^18^^,^^19^^,^^20^ Despite these advances, effective treatment of PD remains a major clinical challenge.

Mitochondrial transplantation has recently emerged as a promising alternative therapeutic strategy for PD.^21^^,^^22^ Intranasal delivery of mitochondria could improve rotational and locomotor behaviors in PD rat models, which was associated with restoration of mitochondrial function and attenuation of oxidative damage in the lesioned SNc.^23^ In addition, intraperitoneal administration of mitochondria isolated from human mesenchymal stem cells exerted neuroprotective effects against MPTP, 6-hydroxydopamine (6-OHDA), and rotenone toxicity and alleviated dopaminergic neuronal loss in the brains of C57BL/6J mice. Transplanted mitochondria showed anti-inflammatory effects by reducing the expression of proinflammatory cytokines in microglial cells and suppressing microglial activation in the striatum.^24^ However, the mechanisms underlying the therapeutic effects of isolated mitochondria in the above reports have not been elucidated, and, therefore, uncertainty regarding transplantation efficiency and delivery strategies persists.^25^^,^^26^

We previously demonstrated that plasma membrane vesicles (PMVs), a type of miniature cytoplast generated by mechanical extrusion, could be utilized for the transplantation of functional mitochondria.^27^ Mitochondrial DNA (mtDNA)-depleted (Rho0) cells and acetaminophen-damaged HepG2 cells transplanted with functional mitochondria derived from mesenchymal stromal cells were protected from cell death and regained mitotic activity.^27^^,^^28^ Compared with approaches using isolated mitochondria, PMV-encapsulated mitochondria sustain less damage during preparation and can be delivered into recipient cells through direct membrane fusion, thereby avoiding endosomal entrapment.

In the present study, we investigated the effects of mitochondrial transplantation on senescent SH-SY5Y cells. Cellular senescence was induced to exhibit a stable senescence phenotype by treatment with 6-OHDA and rhodamine 6G (R6G). Mitochondrial transplantation was able to restore senescent SH-SY5Y cells to an active cell cycle state, normalize cellular metabolism and energy production, and reduce αSyn expression and aggregation. Furthermore, dopamine production and the activities of enzymes involved in dopamine synthesis were significantly enhanced.

## Results

### Reversal of the senescence phenotype of SH-SY5Y cells by mitochondrial transplantation

Cellular senescence has recently emerged as a key feature of neurodegenerative disorders, and senescent SH-SY5Y cells have been widely used as an *in vitro* model of PD.^29^^,^^30^^,^^31^ To establish this model, SH-SY5Y cells were treated with 6-OHDA (Figure S1), a neurotoxin known to induce oxidative stress and mitochondrial dysfunction, ultimately contributing to Lewy body formation and degeneration of dopaminergic neurons.^32^ However, treatment with 6-OHDA alone induced only a transient senescence phenotype, as evidenced by senescence-associated β-galactosidase (SA-β-gal) staining, the intensity of which decreased after withdrawal of 6-OHDA from the culture medium. Subsequent treatment with R6G stabilized the senescence phenotype, as both the intensity of SA-β-gal staining and suppression of mitotic activity remained constant even after withdrawal of 6-OHDA and R6G (Figure S1). This stable senescence phenotype rendered SH-SY5Y cells highly suitable for investigating the effects of mitochondrial transplantation.

**Figure 1:**
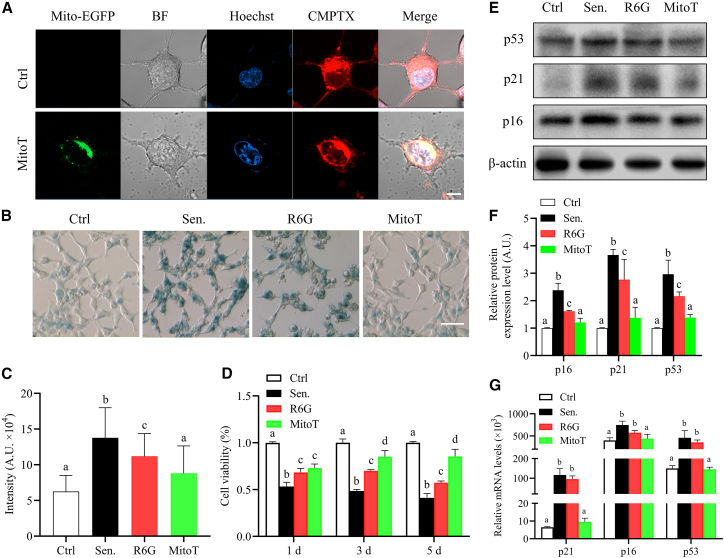
Reversal of the senescence phenotype of SH-SY5Y cells (A) Transplantation of mitochondria (mito-GFP) from Ad293 cells into senescent SH-SY5Y cells (stained with CMPTX). Blue: Hoechst33342. Scale bars, 10 μm. (B) SA-β-gal staining of SH-SY5Y cells. Ctrl, normal SH-SY5Y cells; Sen., senescent SH-SY5Y cells; R6G, PMVs were prepared from Ad293 cells treated with rhodamine 6G for 12 h, which damaged mitochondrial function; MitoT, mitochondrial transplantation via PMVs from normal Ad293 cells. Scale bars, 50 μm. (C) Statistical analysis of SA-β-gal staining intensity in (B). (D) Cell viability was evaluated using a CCK-8 kit at days 1, 3, and 5 post fusion. (E) Western blotting analysis of the expression of cell cycle regulation genes. (F) Semi-quantitative analysis of the protein band intensity in (E). (G) Real-time RT-qPCR analysis of the transcript levels of p16, p21, and p53. *n* = 3. Bars denoted with a different letter on top are significantly different (*p* < 0.05).

Consistent with our previous studies,^27^^,^^28^ mitochondria were delivered into senescent SH-SY5Y cells through fusion with PMVs mediated by the fusogenic vesicular stomatitis virus glycoprotein G (Figure 1A). Mitochondrial transplantation markedly reduced SA-β-gal staining (Figures 1B and 1C) and restored normal cellular morphology and mitotic activity (Figures 1B and 1D). Furthermore, the expression of cell cycle regulatory genes was analyzed by quantitative reverse-transcription PCR (RT-qPCR) and western blotting. In agreement with the SA-β-gal staining and mitotic activity results, mitochondrial transplantation significantly downregulated the senescence-associated cell cycle regulators p16, p21, and p53 (Figures 1E–1G). Importantly, when mitochondrial function within PMVs was disrupted by pretreatment with R6G before fusion, these restorative effects were completely abolished, indicating that functional mitochondria delivered from PMVs are essential for reversal of the senescence phenotype in SH-SY5Y cells.

We further examined the expression of apoptosis-related genes in senescent SH-SY5Y cells. The results showed that the expression of the antiapoptotic genes like *Bcl-2* and *Bcl-*x was significantly reduced, whereas that of the proapoptotic gene *BAX* dramatically increased (Figure S2). However, mitochondrial transplantation completely reversed this tendency of cell death in senescent SH-SY5Y cells.

### Mitochondrial transplantation restored normal glucose metabolism in SH-SY5Y cells

**Figure 2:**
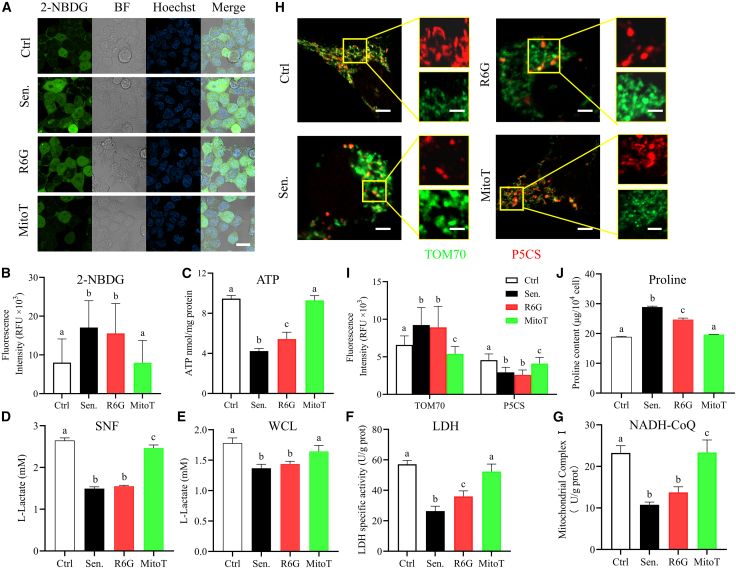
Normalization of glucose metabolism in senescent SH-SY5Y cells (A and B) Measurement of glucose uptake using 2-NBDG two days post fusion. Scale bars, 20 μm. (C) Colorimetry measurement of the ATP content. (D and E) Colorimetric measurement of the extracellular and intracellular lactate content. WCL, whole cell lysate; SNF, supernatant fraction. (F) Measurement of the activity of lactate dehydrogenase (LDH). (G) Measurement of the activity of NADH-CoQ. (H) Immunostaining and confocal microscopy analysis of the expression of P5CS and TOM70. Scale bars, 5 μm. and 2 μm (inset). (I) Measurement of P5CS and TOM70 fluorescence intensity. (J) Colorimetry measurement of the proline content; *n* = 3. Bars denoted with a different letter on top are significantly different (*p* < 0.05).

Dysregulated glucose metabolism is a major characteristic of cellular senescence.^33^^,^^34^ Consistent with this phenomenon, senescent SH-SY5Y cells exhibited increased glucose uptake, accompanied by reduced ATP content, lactate production, lactate dehydrogenase activity, and CI (NADH/CoQ) activity (Figures 2A–2G). Fusion with PMVs carrying functional mitochondria, and not R6G-treated PMVs containing dysfunctional mitochondria, significantly reduced glucose uptake while restoring ATP levels. In parallel, lactate production and lactate dehydrogenase activity returned to near-normal levels after mitochondrial transplantation. These findings indicate that glucose metabolism is severely disrupted in senescent SH-SY5Y cells, with marked impairment of both glycolytic activity and oxidative phosphorylation.

The intracellular distribution of pyrroline-5-carboxylate synthase (P5CS) has been reported to help distinguish between mitochondrial populations involved in oxidative ATP synthesis and reductive biosynthesis of macromolecular precursors required for cellular maintenance.^35^^,^^36^ In senescent SH-SY5Y cells, mitochondrial P5CS predominantly displayed punctate structures, whereas in control cells and cells receiving mitochondrial transplantation via PMVs, P5CS exhibited a mainly filamentous distribution (Figures 2H and 2I). Consistent with these morphological changes, reductive metabolism, reflected by increased proline synthesis, was significantly elevated in senescent cells and subsequently reduced after mitochondrial transplantation (Figure 2J). These results suggest that the balance between oxidative and reductive metabolism is disrupted in senescent SH-SY5Y cells.

### Mitochondrial transplantation attenuated the senescence-associated secretory phenotype

**Figure 3:**
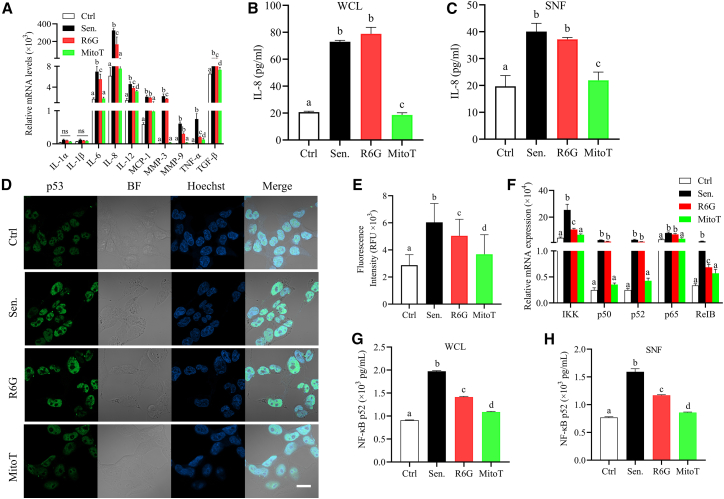
Inhibition of inflammation in senescent SH-SY5Y cells (A) RT-qPCR measurement of inflammatory cytokines two days post fusion. (B and C) Measurement of the extracellular and intracellular IL-8 contents by ELISA. (D) Immunofluorescence staining and confocal microscopy analysis of the p53 expression. Scale bars, 20 μm. (E) Analysis of the fluorescence intensity of p53. (F) RT-qPCR analysis of the expression of NF-κB pathway genes. (G and H) Measurement of the intracellular and extracellular concentrations of p52 component of NF-κB by ELISA. *n* = 3. Bars denoted with a different letter on top are significantly different (*p* < 0.05). WCL, whole cell lysate; SNF, supernatant fraction

Senescence-associated secretory phenotype (SASP) is another defining feature of cellular senescence.^33^^,^^34^ RT-qPCR analysis revealed dramatic upregulation of multiple inflammatory cytokines in senescent SH-SY5Y cells, particularly *IL-6*, *IL-8*, *MMP-3*, *MMP-9*, and *TNF-α*, all of which were significantly downregulated after mitochondrial transplantation (Figure 3A). Intracellular and extracellular IL-8 levels were further quantified using enzyme-linked immunosorbent assay (ELISA), which corroborated the RT-qPCR findings (Figure 3B).

Moreover, two major inflammatory signaling pathways, p53 and NF-κB, were examined. Immunofluorescence staining (Figure 3D), together with RT-qPCR and western blot analyses (Figures 1E–1G), consistently demonstrated activation of the p53 pathway in senescent SH-SY5Y cells, whereas mitochondrial transplantation markedly suppressed this pathway. Similarly, components of the NF-κB signaling pathway, including p52, were significantly downregulated after mitochondrial transplantation (Figures 3F and 3G).

### Mitochondrial transplantation re-established normal mitochondrial function

Senescent SH-SY5Y cells were generated by treatment with 6-OHDA and R6G, both of which are known to induce oxidative stress and impair oxidative phosphorylation,^30^^,^^32^ ultimately leading to mitochondrial dysfunction. Therefore, we investigated the effects of mitochondrial transplantation on mitochondrial functions.

**Figure 4:**
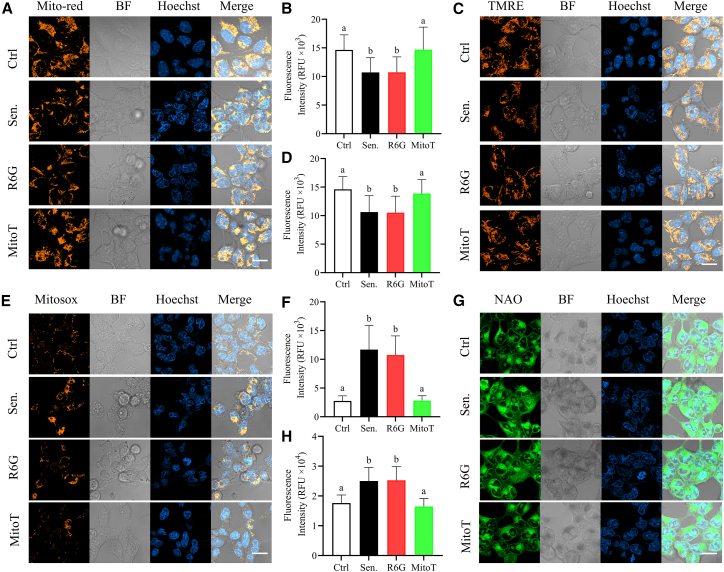
Normalization of mitochondrial functions in senescent SH-SY5Y cells (A, C, E, and G) Mitochondrial shape, membrane potential, ROS levels, and cardiolipin content were evaluated by fluorescence staining with Mito-Tracker Red (A), TMRE (C), Mito-SOX (E), and NAO (G) respectively, two days post fusion. (B, D, F, and H) Quantitative analysis of the abovementioned fluorescence intensity. Scale bars, 20 μm. *n* = 3 (about 50 cells were included in the analysis in each experimental condition). Bars denoted with a different letter on top are significantly different (*p* < 0.05).

Relative mitochondrial content was assessed using MitoTracker Red staining, which demonstrated a marked reduction in total mitochondrial mass in senescent SH-SY5Y cells (Figures 4A and 4B; Figure S7). In addition, tetramethylrhodamine ethyl ester (TMRE) staining revealed a substantial decrease in mitochondrial membrane potential in the senescent cells (Figures 4C and 4D). Conversely, mitochondrial oxidative damage was dramatically increased after treatment with 6-OHDA and R6G (Figures 4E and 4F). Cardiolipin has previously been reported to accumulate in senescent human fibroblasts, while other phospholipid species remained largely unchanged.^36^ Consistent with these findings, cardiolipin levels were significantly elevated in senescent SH-SY5Y cells (Figures 4G and 4H). Importantly, mitochondrial transplantation increased the mitochondrial content and membrane potential while reducing oxidative damage and cardiolipin accumulation (Figure 4), thereby restoring mitochondrial function toward a normal physiological state in SH-SY5Y cells.

### Mitochondrial transplantation restored the formation of a normal network structure

**Figure 5:**
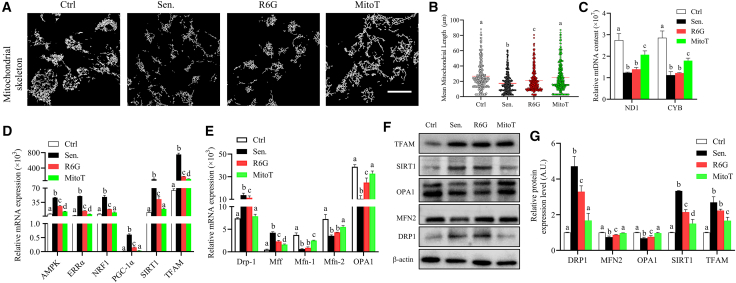
Normalization of mitochondrial biogenesis, fusion, and fission in senescent SH-SY5Y cells (A and B) Analysis of the mitochondrial length and morphology using ImageJ. Scale bars, 20 μm. (C) Quantitative PCR analysis of mitochondrial DNA amount. *β-actin* was used for normalization. (D) RT-qPCR measurement of the expression of genes related to mitochondrial biogenesis. (E) RT-qPCR measurement of the expression of genes related to mitochondrial fusion and fission. (F) Western blotting of the expression of genes related to mitochondrial biogenesis. (G) Quantitative analysis of the protein band intensity in (F); *n* = 3. Bars denoted with a different letter on top are significantly different (*p* < 0.05).

Mitochondrial quality was assessed by analyzing mitochondrial morphology, mtDNA content, mitochondrial fusion, and fission. The results showed that mitochondria predominantly exhibited a fragmented punctate morphology in senescent SH-SY5Y cells. Mitochondrial transplantation restored the elongated interconnected mitochondrial network characteristic of healthy cells (Figures 5A and 5B). mtDNA content was quantified by RT-qPCR analysis of the mitochondrial genes *ND1* and *CYB*, both of which were significantly increased after mitochondrial transplantation (Figure 5C). Interestingly, genes associated with mitochondrial biogenesis were upregulated in senescent SH-SY5Y cells (Figures 5D and 5G), likely reflecting a compensatory response to mitochondrial dysfunction. Consistent with the observed mitochondrial fragmentation, the mitochondrial fission factors *Drp1* and *MFF* were upregulated, whereas the fusion-related factors *Mfn1*, *Mfn2*, and *OPA1* were significantly downregulated in the senescent cells (Figures 5E–5G).

To trace the fate of transplanted mitochondria and evaluate mitochondrial heteroplasmy, the mitochondrial *mt-ND5* was amplified and sequenced to determine the single-nucleotide polymorphism (SNP) variation at days 1, 3, and 5 after fusion. The proportion of transplanted mtDNA increased from approximately 17% (A:G ratio) on day 1 to approximately 100% by day 5 (Figure S6A). Furthermore, the *mt-ND5* SNP was still detected in three independent clones generated approximately 1 month after mitochondrial transplantation (Figure S6B). These findings demonstrate that exogenous mitochondria derived from Ad293 cells were able to stably integrate and persist within SH-SY5Y recipient cells.

**Figure 6:**
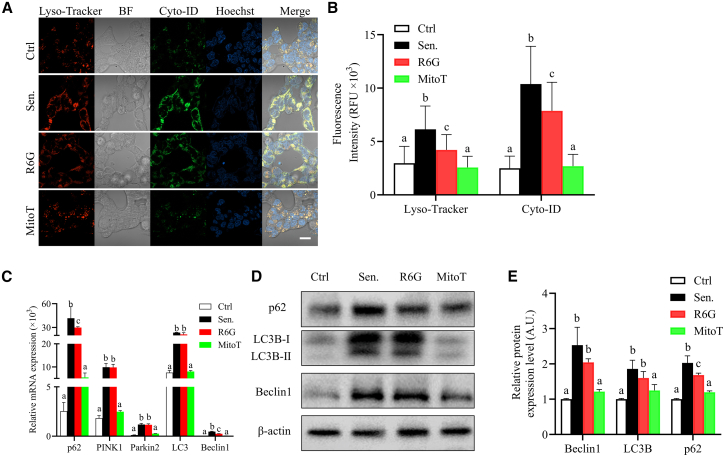
Normalization of autophagy in senescent SH-SY5Y cells (A) Autophagy flux was evaluated by fluorescence staining with LysoTracker (Red) and Cyto-ID (Green) in the presence of chloroquine (20 μM) two days post fusion. Scale bars, 20 μm. (B) Analysis of the fluorescence intensity of LysoTracker and Cyto-ID. (C) RT-qPCR measurement of the expression of genes related to autophagy. (D) Western blotting of the expression of genes related to autophagy. (E) Quantitative analysis of the protein band intensity in (D). *n* = 3. Bars denoted with a different letter on top are significantly different (*p* < 0.05).

To investigate the fate of endogenous mitochondria after transplantation, the autophagic flux was evaluated by Cyto-ID and LysoTracker staining in the presence of chloroquine (Figures 6A and 6B). Autophagy activity was markedly elevated in senescent SH-SY5Y cells but returned to near-normal levels after mitochondrial transplantation. Similar result was observed in the absence of chloroquine, except that fluorescence intensity was significantly reduced (Figure S8). Moreover, we measured the expression of key autophagy-related genes in SH-SY5Y cells (Figures 6C–6E). Consistent with the fluorescence staining results, the mRNA and protein expressions of *p62*, *PINK1*, *Parkin2*, *LC3*, and *Beclin1* were significantly increased in senescent SH-SY5Y cells but returned to near-normal levels after mitochondrial transplantation.

### Mitochondrial transplantation improved the dopaminergic function of SH-SY5Y cells

**Figure 7:**
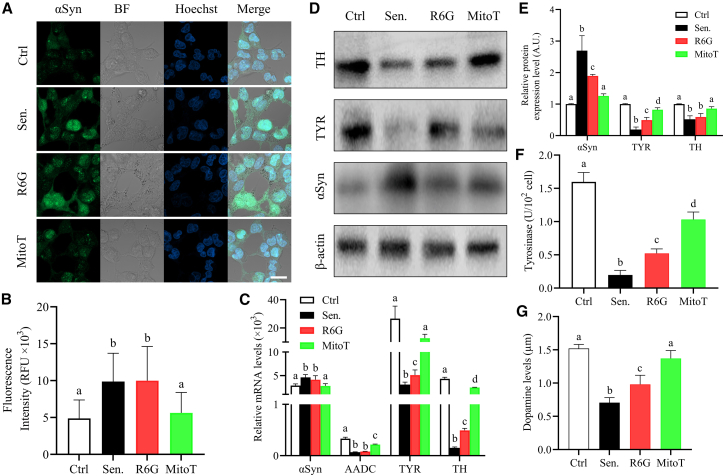
Normalization of αSyn expression and dopamine synthesis in senescent SH-SY5Y cells (A) Immunofluorescence staining and confocal microscopy analysis of the expression of αSyn two days post fusion. Scale bars, 20 μm. (B) Quantitative analysis of the αSyn fluorescence intensity. (C) RT-qPCR measurement of the expression of αSyn, AADC, TYR, and TH genes related to dopamine synthesis. (D) Western blotting of the expression of αSyn, AADC, TYR, and TH genes. (E) Quantitative analysis of the protein band intensity in (D). (F) Measurement of the proline content. (G) Measurement of the intracellular dopamine concentration; *n* = 3. ars denoted with a different letter on top are significantly different (*p* < 0.05).

Finally, we investigated the effects of mitochondrial transplantation on the dopaminergic properties of SH-SY5Y cells. Immunofluorescence staining showed that Syn levels significantly increased in the senescent SH-SY5Y cells (Figures 7A and 7B). Consistently, RT-qPCR and western blot analyses confirmed significant upregulation of αSyn expression in senescent SH-SY5Y cells (Figures 7C–7E). In contrast, mitochondrial transplantation downregulated αSyn expression.

We then measured the expression of genes involved in dopamine production, including *AADC*, *TYR,* and *TH*. Expression of all three genes was significantly decreased at both mRNA and protein levels in senescent SH-SY5Y cells (Figures 7C–7E). In addition, tyrosinase activity was found to reduce to approximately 10% of that observed in normal cells (Figure 7F). After mitochondrial transplantation, *AADC*, *TYR*, and *TH* were significantly upregulated, accompanied by the restoration of tyrosinase activity and a substantial increase in dopamine production (Figure 7G).

## Discussion

The neuroblastoma SH-SY5Y cell line is widely used as an *in vitro* model in PD research.^37^ To induce PD-like pathological features using pharmacological approaches, SH-SY5Y cells are commonly treated with high levels of 1-methyl-4-phenylpyridinium, the active metabolite of MPTP, 6-OHDA, or rotenone, all of which trigger oxidative stress and ultimately lead to cell death.^38^ In contrast, in the present study, SH-SY5Y cells were exposed to relatively low levels of 6-OHDA and R6G, which induce oxidative stress and inhibit mitochondrial oxidative phosphorylation, respectively. The combined effect of 6-OHDA and R6G generated a stable senescence phenotype, characterized by enhanced SA-β-gal staining, cell cycle arrest, and upregulation of senescence-associated cell cycle regulators (Figure 1; Figures S1 and S2). Notably, treatment with 6-OHDA alone induced only transient cellular senescence (Figure S1), whereas the addition of R6G was essential for establishing a stable senescence phenotype, which served as a prerequisite for evaluating the anti-senescence effects of mitochondrial transplantation. Importantly, treatment with 6-OHDA and R6G did not induce overt cell death but instead resulted in persistent cell cycle arrest (Figure S1). These findings are consistent with those of recent studies demonstrating that neurons in SNc exhibit increased senescence-associated characteristics,^39^^,^^40^ suggesting that dopaminergic neurons in PD may not directly undergo apoptosis, but rather enter a senescent state that promotes inflammatory injury in neighboring cells and contributes to neuronal loss.^41^ In agreement with this concept, treatment with 6-OHDA and R6G induced a fragile, apoptosis-prone state characterized by an increased expression of proapoptotic genes and reduced expression of antiapoptotic genes (Figure S3). More importantly, mitochondrial transplantation completely reversed the senescence phenotype and enhanced the antiapoptotic capacity of SH-SY5Y cells.

Mitochondrial transplantation has been widely used in the treatment of various disorders, including neurological diseases.^42^ For example, McCully et al. reported that direct injection of mitochondria into ischemic myocardium reduced infarct size and myocardial cell death while improving cardiac function after ischemic injury.^43^ More recently, mitochondrial transplantation has shown considerable promise for the treatment of PD.^23^^,^^24^ Transplanted mitochondria were reported to exert anti-inflammatory effects by reducing proinflammatory cytokine expression in microglial cells and suppressing microglial activation in the striatum.^24^ Notably, most previously reported mitochondrial transplantation approaches have relied on purified mitochondria, a process that inevitably disrupts the native branched mitochondrial network into fragmented punctate structures. This mechanical disruption may be detrimental because membrane damage can permit uncontrolled exchange between the mitochondrial matrix and surrounding solution, thereby dissipating the proton gradient and eventually leading to a severe loss of membrane potential.^44^^,^^45^

Recently, the mechanisms underlying the uptake and release of naked mitochondria have been elucidated.^46^ Extracellular mitochondria present in the culture medium are internalized by recipient cells primarily through fluid-phase endocytosis. However, only a small proportion (approximately 9%) of the internalized mitochondria can escape the endosomal compartment and subsequently fuse with the endogenous mitochondrial network. In our experimental setting, when naked mitochondria were incubated with senescent SH-SY5Y cells, no significant change was detected in the intensity of SA-β-gal staining (L.X. and C.W., unpublished data). Similarly, Dong et al. reported that naked mitochondria failed to reverse the senescence phenotype of fibroblasts and human umbilical vein endothelial cells induced by high-glucose conditions.^47^ In contrast to approaches using purified mitochondria, our previous studies have demonstrated that PMVs can be effectively utilized for the transplantation of functional mitochondria.^27^^,^^28^ Encapsulation within PMVs minimizes mitochondrial damage during preparation and enables mitochondrial delivery into recipient cells through direct membrane fusion, thereby avoiding endosomal sequestration. Of note, components in PMVs including proteins, RNAs, and organelles other than mitochondria might also contribute to the anti-senescence effects.

Our findings demonstrated that the transplantation of high-quality mitochondria restored the balance between oxidative and reductive metabolism (Figure 2), potentially through modulation of the relative proportions of the two mitochondrial populations distinguished by P5CS distribution.^48^^,^^49^ Interestingly, glycolytic activity was also markedly suppressed despite the apparent increase in glucose uptake. One possible explanation is that glucose flux was redirected toward the pentose phosphate pathway, generating NADPH to counteract oxidative stress in senescent SH-SY5Y cells.

Mitochondrial transplantation also restored multiple aspects of mitochondrial function, including mitochondrial content (Figures 4A and 4B; Figures 5A–5C), mitochondrial membrane potential (Figures 4C and 4D), and reductions in oxidative damage and cardiolipin accumulation (Figures 4E–4H). In addition, mitochondrial quality control was probably improved, as indicated by the increased expression of mitochondrial fusion-related genes and reduced expression of fission-related genes (Figures 5E–5G). Although genes associated with mitochondrial biogenesis were upregulated in senescent SH-SY5Y cells (Figure 5D), this likely represented a compensatory response to mitochondrial dysfunction and reduced mitochondrial abundance (Figures 4A and 4B; Figures 5A–5C). Nevertheless, effective mitochondrial biogenesis may not have occurred because the mtDNA content was actually decreased in senescent SH-SY5Y cells (Figure 5C).

SNP analysis of *mt-ND5* unequivocally demonstrated stable colonization of exogenous mitochondria within recipient SH-SY5Y cells (Figures S4 and S5). Notably, the proportion of exogenous mitochondria gradually increased to nearly 100%, suggesting progressive elimination of endogenous mitochondria through autophagic processes. However, the autophagic activity was significantly higher in senescent SH-SY5Y cells than in control cells (Figure 7). This could be explained by impaired autophagic flux resulting from insufficient ATP levels in senescent SH-SY5Y cells. Transplantation of functional mitochondria likely restored cellular energy supply, thereby enabling completion of the stalled autophagic process. In addition, lysosomal degradative function might have been impaired in senescent cells (L.X. and C.W., unpublished data). Therefore, transplantation of both intact mitochondria and lysosomes via PMVs was necessary to relieve the stalled autophagic flux. Nonetheless, elimination of damaged mitochondria by autophagy would consequently reduce the sources of inflammatory signaling in senescent SH-SY5Y cells (Figure 3).

Interestingly, mitochondrial transplantation markedly reduced both the expression and aggregation of αSyn (Figures 7A–7E), one of the principal pathological hallmarks of PD.^7^ In parallel, the expression of genes essential for dopamine biosynthesis, including *AADC*, *TYR*, and *TH*, was significantly decreased in senescent SH-SY5Y cells (Figures 7C–7E). More importantly, mitochondrial transplantation substantially restored tyrosinase activity and enhanced dopamine production (Figure 7G).

Collectively, these findings demonstrate that PMVs provide a versatile platform for the transplantation of intact and functional mitochondrial networks. This strategy may have broad therapeutic potential for diseases associated with mitochondrial dysfunction and aging. For example, Dong et al. demonstrated that transdermal mitochondrial transplantation from enucleated mesenchymal stem cells to fibroblasts and endothelial cells in diabetic rats promoted chronic wound healing by reversing hyperglycemia-induced cellular senescence.^47^ Furthermore, targeted mitochondrial delivery to the substantia nigra through stereotaxic intracranial administration has also been proposed as a potential therapeutic approach.^50^ Nevertheless, because of the relatively large size of PMVs and the structural complexity of neural tissues, substantial technical challenges must still be overcame before this strategy can be translated into clinical applications.

### Conclusions

In the present study, we developed an innovative mitochondrial transplantation strategy based on fusion with PMVs. Encapsulation within PMVs protected mitochondria from environmental damage during preparation and delivery, while enabling direct entry into the recipient cells through membrane fusion. Using this approach, mitochondrial transplantation not only reversed the senescence phenotype of SH-SY5Y cells but also restored mitotic activity, improved mitochondrial quality control, attenuated inflammatory responses, reduced αSyn expression, and enhanced dopamine production together with the activities of enzymes involved in dopamine synthesis. However, to more closely recapitulate the pathological features of PD, SH-SY5Y neuroblastoma cells should be differentiated into neuron-like cells before experimentation. In addition, the introduction of a αSyn transgene could increase the expression and promote protein aggregation.

## Material and methods

### Induction of senescent SH-SY5Y cells

The SH-SY5Y cells were cultivated in DMEM medium supplemented with 10% FBS, 1% glutamine, and 1% penicillin and streptomycin. The cells were incubated at 37°C with 5% CO_2_ and saturating humidity. To induce cellular senescence, the cells were cultivated with 10 μM 6-OHDA for three days, and then with 1 μM R6G for another three days. SA-beta-gal staining was then carried out according to a protocol provided by the company (Beyotime, Shanghai, China). Briefly, the cells were fixed at room temperature for 15 min, washed with pre-warmed PBS three times, and then incubated with freshly made staining solution at 37°C for 36 h. The cells were then examined and photographed using a converted microscope equipped with a CCD camera (Zeiss, Oberkochen, Germany). To verify the senescent state of SH-SY5Y cells, their viability was examined using a CCK-8 kit according to the procedure provided by the company (Beyotime).

### Preparation of VSV-G condition medium

Ad293 cells (Agilent, Santa Clara, CA) were seeded in 6-well plates overnight and transfected with 0.8 μg/well of pLV-VSVG plasmid, using the PolyJet reagent (SignaGen Laboratories, Ijamsville, MD) as per the instruction manual. Condition medium was collected at 48 h. Ad293 cells were harvested and lysed by three freeze-thaw cycles. The lysate and conditioned medium were transferred to 2-mL tubes, and cell debris was removed by differential centrifugation at 300, 2,000, and 10,000 g for 10 min at each step. Clear conditioned medium (CM) was then condensed by super centrifugation (Type 100Ti fixed angle, 344619, Beckman Coulter) at 100,000 g for 70 min. About 200 μL of concentrated VSV-G condition medium was collected from 6 mL of debris-free medium. The total protein concentration was then measured using the bicinchoninic acid (BCA) assay (Thermo Scientific, Waltham, MA).

### Mitochondria transplantation into SH-SY5Y cells

The SH-SY5Y cells were seeded overnight and then labeled with CMTPX (CellTracker Red CMTPX, Yeasen Biotechnology, Shanghai, China). The PMVs were prepared from Ad293 cells that carry a stable mito-GFP transgene. The cells were extruded through a filter fitted with a 5-μm membrane, as reported previously.^27^^,^^28^ PMVs (about 100 μL with a total protein concentration of 1 μg/mL) were mixed with concentrated VSV-G condition medium and then added to SH-SY5Y cells in a 96-well plate (about 1 × 10^4^ cells). The medium was aspirated after 30-min incubation, and then fusion solution (0.8 mM citrate buffer, pH 4.5) was added. Fusion induction was carried out for 1 min, and the medium was changed back to normal DMEM culture medium (pH 7.5) for 30 min. Finally, the cells were stained with Hoechst 33342 (Yeasen Biotechnology) and then examined by confocal microscopy (Zeiss 800).

### Real-time RT-qPCR

Total RNA was isolated using TRIzol reagent according to the manufacturer’s protocol. Reverse transcription was carried out using a ToloScript RT EasyMix for qPCR Kit according to the instructions provided by the company (TOLO Biotech, Shanghai, China). Two-Step gDNA Erase-Out Mix was added to 1 μg of total RNA for the removal of residual DNA in a volume of 16 μL at 42°C for 2 min. ToloScript qRT EasyMix was then added to the above reaction mixture for reverse transcription in a volume of 20 μL at 37°C for 15 min. After reverse transcription, the cDNA was diluted with H_2_O into a volume of 100 μL, of which 5 μL was used in quantitative real-time PCR (real-time qPCR), which was carried out in a 96-well plate using the LightCycler480 system (Roche, Rotkreuz Switzerland) with the following thermal conditions: 95°C for 5 min, followed by 40 cycles of 95°C for 20 s and 60°C for 30 s. The reaction for each sample was performed in triplicate. The expression level of *ß-actin* was used for normalization. A cycle threshold of 38 was designated arbitrarily as 1. Sequences of specific primers are provided in the supplemental materials (Table S1).

### Western blotting

The cells were harvested and lysed in RIPA buffer supplemented with PMSF (1 mM, Beyotime) and phosphatase inhibitors (MCE, Birmingham, AL). The cells were pipetted repeatedly and incubated on ice for 10 min. The cells were centrifuged at 4°C and 12,000 rpm for 30 min. The supernatant was collected, and the protein concentration was determined using a BCA kit. The samples were mixed with 5× SDS loading buffer and heated at 100°C for 5 min. An equal amount of protein was loaded for electrophoresis. Proteins in SDS PAGE were transferred to PVDF (polyvinylidene fluoride) films, blocked for 1 h with western blot blocker buffer (QuickBlock, Beyotime), washed with TBST three times, and then incubated with appropriate antibodies overnight at 4°C. After washing, the film was incubated with secondary antibodies in 5% defatted milk solution at room temperature for 1 h. After washing three times, the membrane was developed using the enhanced chemiluminescence (ECL) solution (ChemStudio SA, Thermo Fisher, Carlsbad, CA). The protein band intensity was quantified using Image-Pro Plus software (Media Cybernetics, Rockville, MD). The cells treated with staurosporine (STS, 10 μM, Aladdin, Shanghai, China) or TNF-α (1000×, Beyotime) were used as positive controls. Antibody information is provided in the supplemental materials (Table S2).

### SNP analysis of mtDNA

After fusion, the cells were lysed, and the mt-DNA gene was amplified by PCR at days 1, 3, and 5 with primer pairs reported previously^51^: mt-ND5, 5′-ACTACTATAACCACCCTAACCCTG-3′ (F), 5′-TTAGGGAGAGCTGGGTTGTTTGG-3′ (R). PCR products were sequenced for SNP determination. Further, three clones of SH-SY5Y cells were generated one month after mitochondrial transplantation. SNP analysis of the mt-ND5 gene was carried out as described above. To ensure the identity of the recipient cells, SH-SY5Y cells were stably transfected with an EGFP gene. This could be identified by the green fluorescence and by PCR analysis using the following primer pairs: EGFP, 5′-CCCGACAACCACTACCTGAG-3′ (F), 5′-GTCCATGCCGAGAGTGATCC-3′ (R). PCR products were sequenced by Sangon Biotech (China).

### Detection of autophagy

To measure autophagy flux, SH-SY5Y cells transplanted with or without mitochondria were stained with MitoTracker-Red (200 nM, Beyotime) or LysoTracker red (50 nM, Enzo Life Science, Farmingdale, NY) together with CYTO-ID Green (Enzo Life Science) in the presence or absence of chloroquine (CQ, 60 μM) at 37°C for 20 min, 48 h post fusion. The R6G group was used as the control. After washing, the cells were examined by confocal microscopy. Autophagy was further evaluated by western blot analysis as described above. Antibody information is provided in the supplemental materials (Table S2).

### Immunofluorescence staining

After fixation with 4% paraformaldehyde (P1110, Solarbio, Santiago, CA), the cells were permeabilized with Triton X-100 (P0096, Beyotime). The cells were blocked with the QuickBlock Blocking Buffer (P0252, Beyotime) for 30 min and then incubated with a primary antibody overnight at 4°C. After washing with PBS, the cells were incubated with a corresponding secondary antibody at room temperature. After Hoechst 33342 (P0133, Beyotime) staining, images were captured and analyzed using an inverted fluorescence microscope (ZEISS). Antibody information is provided in the supplemental materials (Table S3).

### Measurement of IL-8 and NF-κB p52 by ELISA

The p52 of NF-κB and the IL-8 concentrations were determined using a corresponding ELISA kit (Huabio, Hangzhou, China) according to the manufacturer’s instructions. Absorbance at 450 nm was measured using a microplate reader (BioTek, Winooski, VT). The concentrations of p52 and IL-8 were determined according to a standard curve generated by using the standard provided by the company.

### Measurement of metabolic activities

Glucose uptake was measured using 2-NBDG (MCE) at a final concentration of 200 μM. The cells were incubated with 2-NBDG at 37°C for 30 min in PBS. After washing, images were captured using an inverted fluorescence microscope (ZEISS). The fluorescence intensity was analyzed using ZEN 2 software.

The concentration of ATP was measured using an ATP detection kit (S0026, Beyotime) according to the manufacturer’s instructions. After centrifugation, the cell debris was removed, and the supernatant was added to the substrate solution. Luminescence was recorded using an Illuminometer (PCE Instruments, Jupiter, FL) with an integration time of 10 s per well. The ATP concentration was determined according to a standard curve generated by using the standard provided by the company.

The activity of mitochondrial complex I (NADH-CoQ reductase) in the cell was determined using a corresponding kit (EBCK834M, Elabscience, Wuhan, China) according to the manufacturer’s instructions. Absorption at 340 nm was measured using a microplate reader (BioTek).

Lactate dehydrogenase (LDH) activity was determined using an LDH activity assay kit (EBCK766M, Elabscience) according to the manufacturer’s instructions. Absorption at 450 nm was obtained using a microplate reader (BioTek).

Lactate concentration was determined using an L-Lactate Assay Kit (S0208S, Beyotime) according to the manufacturer’s instructions. Absorption at 450 nm was measured using a microplate reader (BioTek).

The proline concentration was determined using a Proline (Pro) Content Assay Kit (BC0290, Solarbio, Beijing, China) according to the manufacturer’s instructions. Absorption at 520 nm was measured using a microplate reader (BioTek).

### Evaluation of mitochondrial functions

The mitochondrial shape, membrane potential, and cardiolipin content were examined by staining with Mito-Tracker red (200 nM, Thermo Fisher Scientific, Waltham, MA), TMRE (10 nM, Abcam, Waltham, MA), and acridine orange 10-nonyl bromide (NAO, 1 μM, MCE), respectively. Briefly, the cells seeded in an 8-chambered cover glass were incubated with dyes for about 30 min in the dark. After washing, the cells were imaged using a confocal microscope (ZEISS).

### Measurement of tyrosinase activity

The tyrosinase activity was assayed according to the protocol provided by the company (AKAM010C, BoxBio, Beijing, China). Briefly, the cells (1 × 10^4^) were harvested and dispersed in the abstraction solution. The cells were sonicated on ice-water (200 W, 3 s, and repeated 30 times) and then centrifuged (12,000 g) at 4°C for 20 min. Supernatant was collected and added to a microplate. The sample was measured at 475 nm for 10 s (A1) and then measured at 37°C for 190 s (A2). The tyrosinase activity was calculated using the following equation: tyrosinase (U)/10^4^ cells = 180.18 × (A2-A1).

### Measurement of dopamine by HPLC

The dopamine levels were measured according to a previously reported method.^52^ For high-performance liquid chromatography (HPLC) sample preparation, the cells were lysed by three freeze-thaw cycles. The protein content of the supernatant was determined by BCA assay. The supernatant was then stored at −80°C until use.

HPLC analysis for the dopamine of the supernatants was carried out using Agilent 1260 Infinity ll system with electrochemical detection (Agilent Technologies, Santa Clara, CA), together with an Uniget C-18 reverse-phase microbore column as the stationary phase (BASi, cat no. 8949). The mobile phase consisted of buffer (0.1 M monochloro acetic acid, 0.5 mM Na-EDTA, 0.15 g/L Na-octylsulfonate, and 10 nM sodium chloride, pH 3.1 [all chemicals were from Sigma, Saint Louis, MO]), acetonitrile, and tetrahydrofuran (all solvents from Fisher Scientific, Waltham, MA) in a ratio 94:3.5:0.7. The flow rate was 1.0 mL/min, and the working electrode (Uniget 3-mm glassy carbon, BAS P/N MF-1003) was set at 550 mV vs. Ag/Ag/Cl reference electrode. The detection gain was set to 1.0 nA, the filter was 0.2 Hz, and the detection limit to 20 nA. 5 μL of the sample supernatant was directly injected into the HPLC for analysis. Standard dopamine was used to quantify and identify the peaks on the chromatographs. The retention times for dopamine were approximately 6.3 min under the set conditions.

### Statistical analysis

All statistical analyses were performed using GraphPad Prism 7 (GraphPad Software Inc., San Diego, CA). All data represent the mean ± standard deviation. The experiments were performed independently for at least 3 times, and in each experiment, three biological replicas were included. Three of the typical experimental results were selected for statistical analysis. The results were analyzed using a two-tailed Student’s *t* test and one-way ANOVA with Tukey’s post hoc test. *p* values of <0.05 were considered statistically significant.

## Data and code availability

All data supporting the findings of this study are available within the article and its supplementary information files.

## Acknowledgments

This work was supported by the 10.13039/501100003453Natural Science Foundation of Guangdong (http://gdstc.gd.gov.cn/grant nos. 2019A1515011547, 2023A1515011906, and 2023A1515012586); Guangdong High-Level University Project “Green Technologies for Marine Industries”; 10.13039/100007421Li Ka Shing Foundation (grant no. 2020LKSFG10C); and Scientific Research Initiation Grant (NTF20030 and NTF22025). Informed consent was obtained from all individual participants included in the study. The authors declare that they have not used AI-generated work in this manuscript. The usage of cell lines including Ad293 (AE-H407, Shanghai Yubo Biotechnology, Shanghai, China) and SH-SY5Y (SCSP-5014, China Collection of Cell Lines, Shanghai, China) was approved by the Animal and Human Experiment Ethical Committee of Shantou University in March 4, 2024 (Establishment of a novel mitochondrial transplantation technique, no. 202401007).

## Author contributions

Conceptualization, L.X. and C.-j.W.; investigation, L.X., Y.W., W.W., X.L., and R.D.; methodology, L.X., Y.W., W.W., X.L., R.D., H.Z., A.M., P.S., X.Z., W.X., and C.-j.W.; validation, L.X., Y.W., W.W., X.L., R.D., and C.-j.W.; writing – original draft, L.X. and C.-j.W.; writing – review & editing, L.X., H.Z., A.M., P.S., X.Z., W.X., and C.-j.W.; resources, P.S., X.Z., W.X., and C.-j.W.; supervision, H.Z., A.M., and C.-j.W.; funding acquisition, H.Z., A.M., and C.-j.W.

## Declaration of interests

The authors declare no competing financial interests.

## Supplemental information

Document S1. Figures S1–S8 and Tables S1–S3

Document S2. Article plus supplemental information
