# Supplementary material for: Mitochondrial transplantation reverses the senescence phenotype of SH-SY5Y cells
Source: Mol Ther Adv. 2026 Jun 17;34(3):201788. doi: 10.1016/j.omta.2026.201788 (PMC13343158; doi:10.1016/j.omta.2026.201788)
Supplement: Document S1. Figures S1–S8 and Tables S1–S3 [file mmc1.pdf]

OMTA, Volume *34*

Supplemental information

Mitochondrial transplantation reverses the senescence phenotype of SH-SY5Y cells

Liqun Xu, Yilang Wu, Wanfei Wu, Xiao Li, Ronghao Deng, Haibao Zhu, Aihua Mao, Pingnan Sun, Xin Zhang, Wencan Xu, and Chi-ju Wei

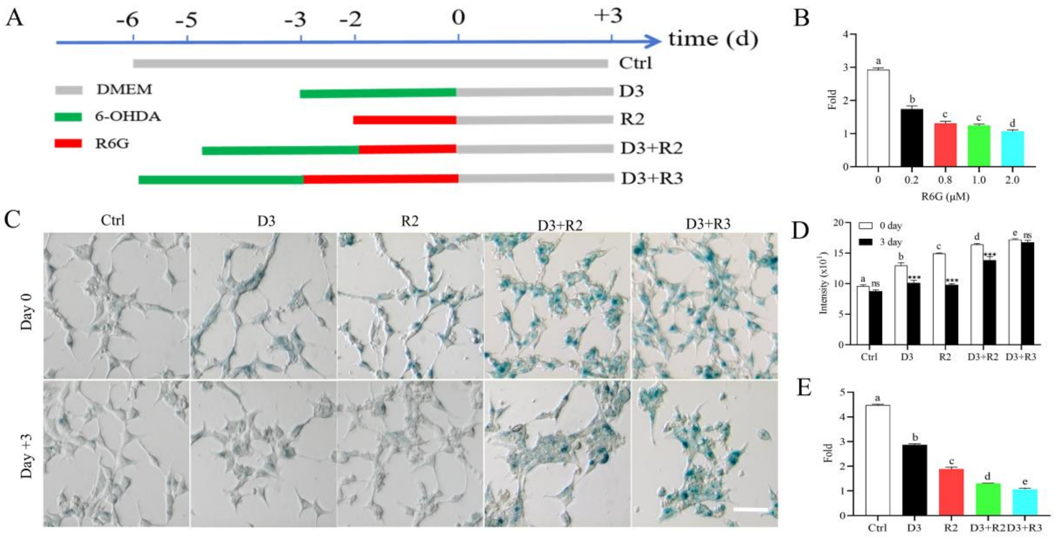

Figure S1 Establishment of a senescent cellular model. (A) SH-SY5Y cells were treated with 6-OHDA and/or R6G for 2-3 days as indicated. The culture medium was placed with normal DMEM for another 3 days. (B) SH-SY5Y cells were treated with different concentration of R6G for 3 days. Cell viability assay was carried out using a CCK-8 kit. (C) SA-β-gal staining of SH-SY5Y cells treated with 6-OHDA and/or R6G as indicated in A. (D) Intensity of SA-β-gal staining. (E) Viability of SH-SY5Y cells in C at day +3. n = 3. Bars denoted with a different letter on top are significantly different ($p < 0.05$).

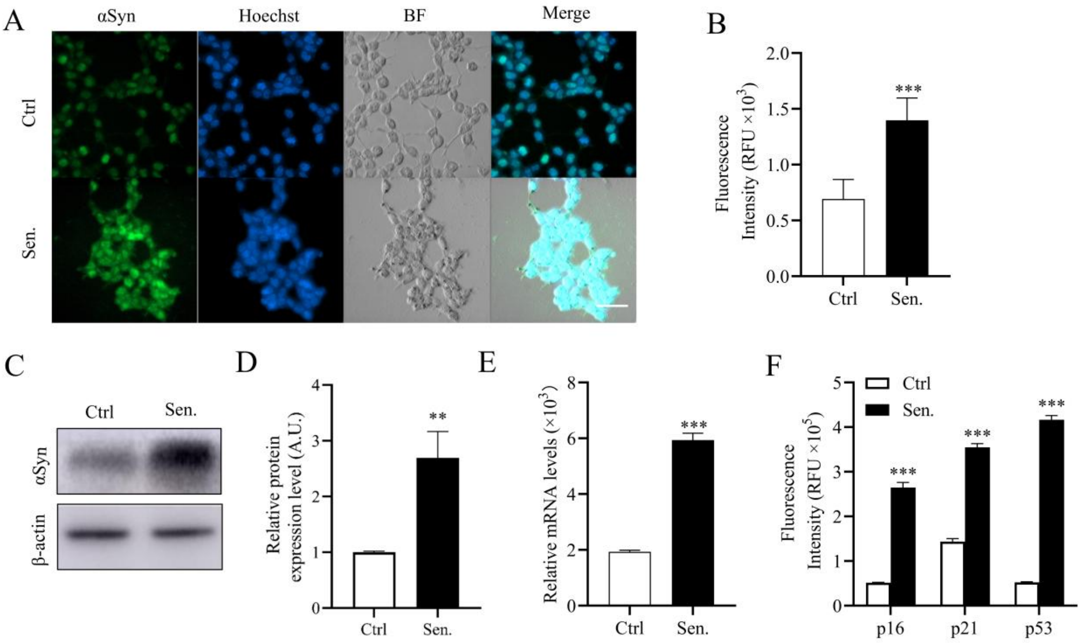

Figure S2 Expression of αSyn and cell cycle control genes. (A) Immunofluorescence staining of α Syn in SH-SY5Y cells after treatment with 6-OHDA and R6G. (B) Fluorescence intensity of A. (C) Western blot analysis of αSyn. (D) Intensity of αSyn protein band in C. (E) mRNA levels of αSyn. (F) mRNA levels of cell cycle control genes. n = 3. Bars denoted with a different letter on top are significantly different (p < 0.05).

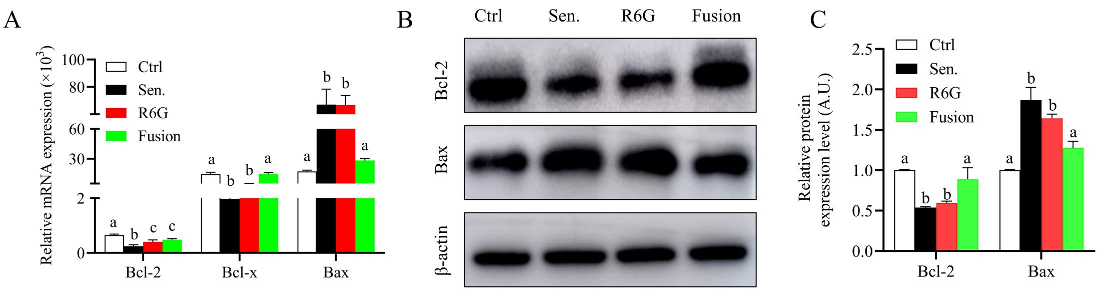

Figure S3 Anti-apoptosis effects of mitochondrial transplantation. (A) mRNA levels of genes essential to apoptosis. (B) Western blot analysis of genes essential to apoptosis. (C) Intensity of Bcl-2 and BAX protein bands. n = 3. Bars denoted with a different letter on top are significantly different ($p < 0.05$).

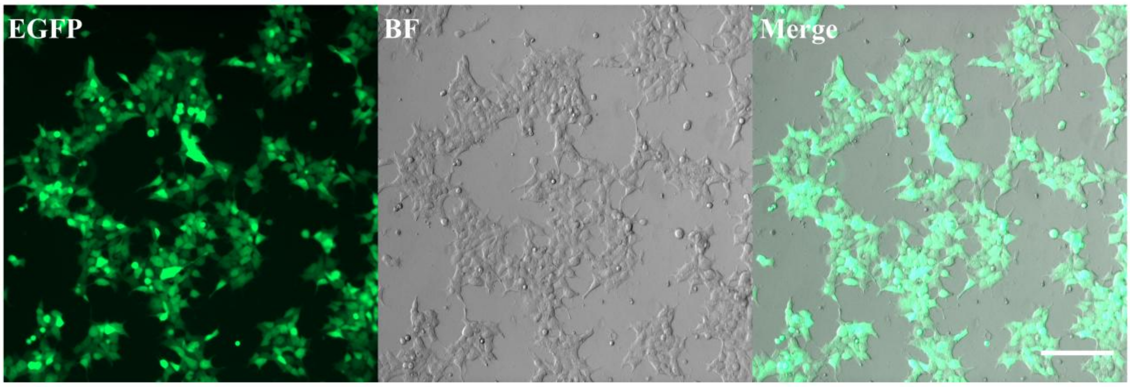

Figure S4 Generation of SH-SY5Y-EGFP cell line. SH-SY5Y cells were transfected with an expression cassette of EGFP. Positive cell line was established by cloning in 96-wells. Scale = 20 μm.

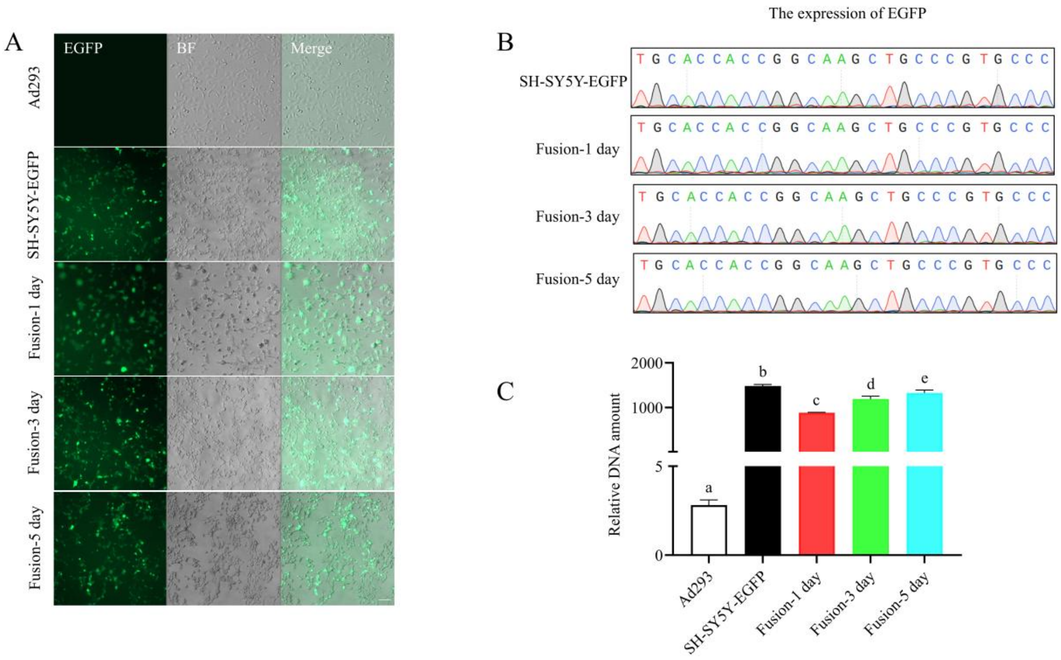

Figure S5 Tracing of the recipient cells. (A) SH-SY5Y-EGFP cells were fused with PMVs. The presence of green fluorescence was traced on day 1, 3 and 5. (B) The presence of EGFP gene was determined by PCR amplification and sequencing. No signal was detected from Ad923 cells. (C) EGFP was amplified by Quantitative real time PCR. n = 3. Bars denoted with a different letter on top are significantly different ($p < 0.05$).

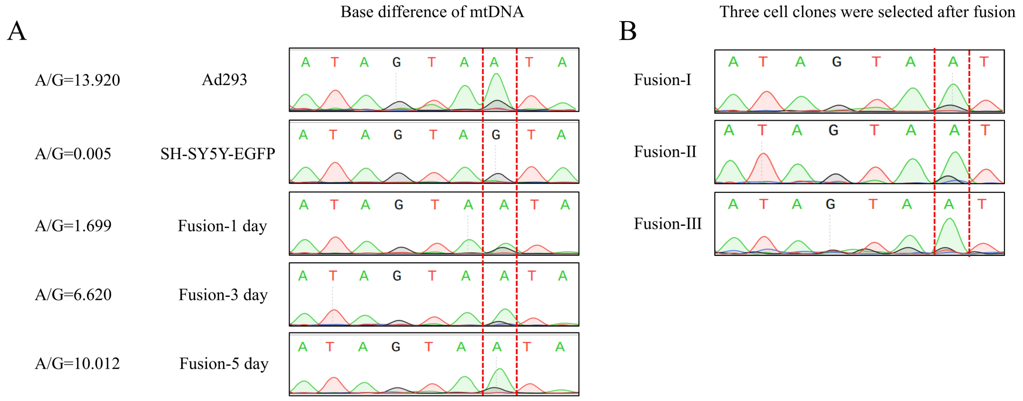

Figure S6 Tracing of exogenous mtDNA by SNP analysis. (A) mt-ND5 was amplified by PCR and SNP site was determined after sequencing. (B) SNP of mt-ND5 was determined in three SH-SY5Y cell clones post-fusion. The SNP site is denoted with dotted lines.

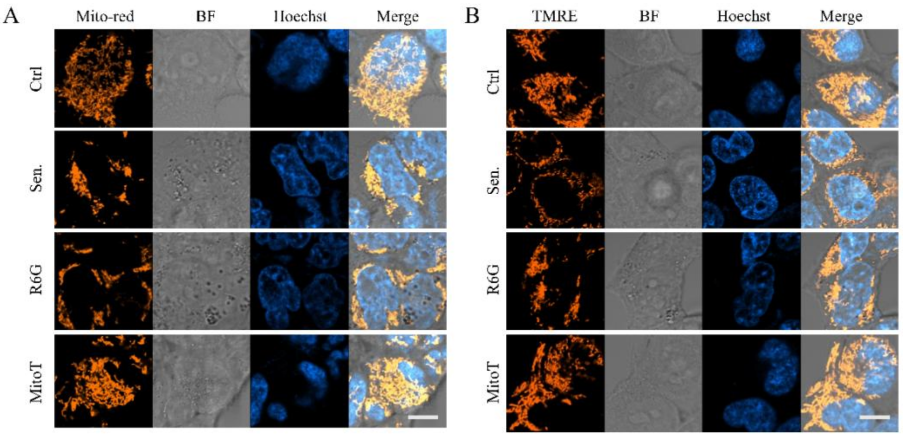

Figure S7 Enlarged representation of Figure 4A/B. Two days post-fusion, mitochondrial shape and membrane potential were evaluated by fluorescence staining with Mito-Tracker Red (A) and TMRE (B). Scale = 10 μm.

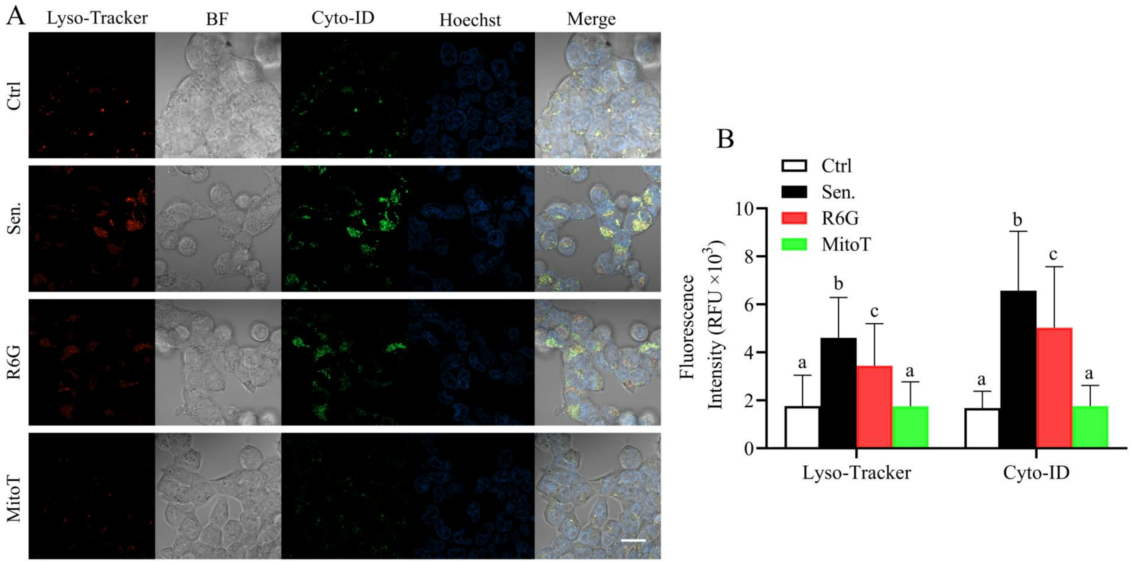

Figure S8 Analysis of autophagy in the absence of CQ. (A) Autophagy flux was evaluated by fluorescence staining with Lysotraker (Red) and Cyto-ID (Green) in the absence of chloroquine (20 μM) two days post-fusion. Scale = 20 μm. (B) Analysis of the fluorescence intensity of Lysotraker and Cyto-ID.

Table S1 Primer pair sequences used in this study

Gene	Forward Primer Seq 5'-3	Reverse Primer Seq 5'-3'
p53	GTACCACCATCCACTACAACTAC	CACAAACACGCAACCTCAAAG
p21	TGGAGACTCTCAGGGTCGAAA	GGCGTTGGAGTGGTAGAAATC
p16	CAGTAACCATGCCCGCATAGA	AAGTTTCCCGAGGTTTCTCAGA
IL-1α	AGTAGCAACCAACGGGAAGG	AAGGTGCTGACCTAGGCTTG
IL-1β	ATGATGGCTTATTACAGTGGCAA	GTCGGAGATTCGTAGCTGGA
IL-6	CCAGTACCCCCAGGAGAAGA	CAGCTCTGGCTTGTTCCTCA
IL-8	AAGACATACTCCAAACCTTTCCACC	CTTCAAAAACTTCTCCACAACCCTC
IL-12	CCTTGTGGCTACCCTGGTCCTC	CTCAGCAGGTTTTGGGAGTGGT
MCP-1	GATCTCAGTGCAGAGGCTCG	TTTGCTTGTCCAGGTGGTCC
MMP-3	GGTTCCGCCTGTCTCAAGAT	GGTTCTGGAGGGACAGGTTC
MMP-9	GGACAAGCTCTTCGGCTTCT	TCGCTGGTACAGGTCGAGTA
TNF-α	CTGGAAAGGACACCATGAGCA	TCTCTCAGCTCCACGCCATT
TGF-β	TGGTGGAAACCCACAACGAA	GAGCAACACGGGTTCAGGTA
Bcl-2	GACTTCGCCGAGATGTCCAG	GAACTCAAAGAAGGCCACAATC
Bcl-x	CGGGCATTCAGTGACCTGAC	TCAGGAACCAGCGGTTGAAG
Bax	ATCAGAACCATCATGGGCTGGACA	AGCCCATCTTCTTCCAGATGGTGA
IKK	TGTACACAGAGTTCTGCCCG	TGCTGAAGTCTCCCCATCTTG
p50	GTGCAGAGGAAACGTCAGAA	GTGGGAAGCTATACCCTGGA
p52	GCGTTGTCAACCTCACCAAC	GAGTCTCCATGCCGATCCAG
p65	CTTCCTGCCCTACAGAGGTC	AAGGCACTTGAGAAGAGGGA
ReIB	ATCAAGGAGAACGGCTTCGG	CAGGGTGACCGTGCTCAG
AMPK	CGGAGCCTTGATGTGGTAGG	AGATGGTGTACTGATGACCTGG
ERRα	GCTACCACTATGGTGTGGCA	TACTCGATGCTCCCCTGGAT
NRF1	GCTAATGGCCTGGTCCAGAT	GGTGACTGCGCTGTCTGATA
PGC-1α	TGGATGAAGACGGATTGCC	TAGCTGAGTGTTGGCTGGTG
SIRT1	TCCTGGACAATTCCAGCCAT	TTTGGATTCCCGCAACCTGT
TFAM	TCAGCATGCTAAAGAGGACGA	TTGTGCGACGTAGAAGATCC

Drp-1	GAGTAAGCCCTGAACCAA	TGATGAACCGAAGAATGAG
Mff	CACCGATTTCTGCACCGGA	CGCAACACAGGTCTGCGATTT
Mfn-1	GGCTAAGAAGGCGATTACTGC	TGCCACCTTCATGTGTCTCC
Mfn-2	ACCGCCACATAGAGGAAG	GCACAGACACAGGAAGGA
OPA1	ATCTGTGGATGCTGAACGCA	GAATCCTGCTTGGACTGGCT
p62	GCTCAGGAGGAGACGATGAC	AGAAACCCATGGACAGCATC
PINK1	ACGTTCAGTTACGGGAGTGG	GGCTAGTCAGGAGGGAAACC
Parkin2	CTGACACCAGCATCTTCCAG	CCAGTCATTCCTCAGCTCCT
LC3	GATGTCCGACTTATTCGAGAGC	TTGAGCTGTAAGCGCCTTCTA
Beclin1	GTATCGACTCATCCCCTGCG	TGAGGAAAACATCCTGCCCC
TH	CAAGTTCGACCCTGACCTGG	GTACTCCACACGGGGAATCG
AADC	AGCCCCTACTTCTTCGCCTA	CGAGCCAGTCCATCATCACA
TYR	GCACCCATTGGACATAACCG	AGAGTCTGGGTCTGAATCTTGT
α-syn	GGCTCCAAAACCAAGGAGGG	CAGAATTCCTTCCTGTGGGGC
β-actin	CCTGGCACCCAGCACAAT	GGGCCGGACTCGTCATAC
ND1	GAAGTCACCCTAGCCATCATTC	GCAGGAGTAATCAGAGGTGTTC
CYB	CTAGGCGACCCAGACAATTATAC	TTAGGGACGGATCGGAGAAT
ND5	ACTACTATAACCACCCTAACCCTG	TTAGGGAGAGCTGGGTTGTTTGG

Table S2 Information of antibodies used for Western blot analysis

	Antibody	Brand	Catalog Number
Primary antibody	Alpha-Synuclein Recombinant Rabbit Monoclonal Antibody	HUABIO	ET7107-31
	Tyrosinase Recombinant Rabbit Monoclonal Antibody	HUABIO	ET1704-18
	Tyrosine Hydroxylase Recombinant Rabbit Monoclonal Antibody	HUABIO	ET1611-12
	TFAM Rabbit mAb	ABclonal	A3173
	SIRT1 Rabbit mAb	ABclonal	A19667
	OPA1 Rabbit pAb	ABclonal	A9833
	Mitofusin 2 Rabbit mAb	ABclonal	A19678
	DRP1 Rabbit mAb	ABclonal	A21968
	Bcl-2 Recombinant Rabbit Monoclonal Antibody	HUABIO	HA721235
	Bax Recombinant Rabbit Monoclonal Antibody	HUABIO	ET1603-34
	p53	SANTA CRUZ	sc-71817
	CDKN1A/P21 Rabbit Polyclonal Antibody	HUABIO	HA500156
	p16INK4a Recombinant Rabbit Monoclonal Antibody	HUABIO	ET1608-62
	HRP Conjugated beta Actin Recombinant Rabbit Monoclonal Antibody	HUABIO	ET1702-67
	SQSTM1/p62 Rabbit mAb	ABclonal	A19700
	LC3B Recombinant Rabbit Monoclonal Antibody	HUABIO	ET1701-65
	Beclin 1 Recombinant Rabbit Monoclonal Antibody	HUABIO	HA721216
Secondary antibody	Goat Anti-Rabbit IgG H&L (HRP)	Abcam	ab6721

Table S3 Information of antibodies used for immunofluorescence staining

	Antibody	Brand	Catalog Number
Primary antibody	TOM70 Monoclonal antibody	Proteintech	66593-1-PBS
	P5CS Polyclonal antibody	Proteintech	17719-1-AP
	p53	SANTA CRUZ	sc-71817
	Alpha-Synuclein Recombinant Rabbit Monoclonal Antibody	HUABIO	ET7107-31
Secondary antibody	Goat anti-Rabbit IgG (H+L) Cross-Adsorbed Secondary Antibody, Alexa Fluor™ 594	Invitrogen	A-11012
	Goat anti-Mouse IgG (H+L) Cross-Adsorbed Secondary Antibody, Alexa Fluor™ 488	Invitrogen	A-11001
	Goat anti-Rabbit IgG (H+L) Cross-Adsorbed Secondary Antibody, Alexa Fluor™ 488	Invitrogen	A-11008
